# The impact of statin therapy on amputation risk in patients with diabetic foot infection: a retrospective cohort study from Jordan

**DOI:** 10.3389/fendo.2026.1922755

**Published:** 2026-09-16

**Authors:** Jana Abdallah, Mera A. Ababneh, Anan S. Jarab, Abeer M. Ayoub, Wasan Khwaileh, Nada A. Alsaleh

**Affiliations:** 1 Department of Clinical Pharmacy, Faculty of Pharmacy, Jordan University of Science and Technology, Irbid, Jordan; 2 Department of Pharmacy Practice, College of Pharmacy, Princess Nourah bint Abdulrahman University, Riyadh, Saudi Arabia

**Keywords:** amputation risk, diabetes, diabetic foot infection, dose–response relationship, retrospective cohort study, statin therapy

## Abstract

**Background:**

Diabetic foot infection (DFI) is a major cause of non-traumatic lower-limb amputation and is associated with poor survival outcomes. Identifying therapies that reduce progression to amputation is critical.

**Objective:**

To evaluate the association between statin therapy and risk of amputation in patients with DFI, including assessment of dose-response effects.

**Methods:**

A retrospective cohort study involving 376 patients presenting with DFI at a university hospital in Jordan between January 2020 and April 2024. The primary outcome was amputation severity (no amputation, minor amputation, major amputation), analyzed using multivariable ordinal logistic regression, adjusting for potential confounders; major amputation incidence was also examined as a secondary binary outcome for absolute risk reduction and number-needed-to-treat calculations.

**Results:**

Moderate-intensity statin therapy was associated with an absolute risk reduction (ARR) of 9.3% (NNT = 11) for major amputation, while high-intensity therapy was associated with an ARR of 14.3% (NNT = 7). In adjusted ordinal logistic regression models, both moderate-intensity (aOR 0.43, 95% CI 0.20–0.74, p = 0.004) and high-intensity statins (aOR 0.43, 95% CI 0.26–0.71, p = 0.001) were associated with lower odds of more severe amputation. A significant dose–response trend was observed, with major amputation rates decreasing from 21.9% in untreated patients to 12.6% and 7.6% in moderate- and high-intensity groups, respectively (p = 0.025). Subgroup analyses showed consistent associations across key clinical subgroups, including patients with advanced Wagner grades, peripheral arterial disease, and neuropathy.

**Conclusion:**

Statin therapy was associated with lower odds of more severe amputation and a lower observed risk of major amputation in patients with diabetic foot infection. An ordered trend in risk reduction was observed across statin intensity levels, but moderate- and high-intensity statins did not differ significantly from one another, so these findings support an association with statin use rather than a confirmed dose-dependent gradient. Further randomized controlled trials are needed to confirm causality and evaluate long-term outcomes.

## 1 Introduction

Diabetes mellitus (DM) is a major and rapidly growing global health concern, affecting an estimated 589 million adults in 2024, with projections indicating an increase to 853 million by 2050 (1). The Middle East and North Africa (MENA) region, of which Jordan is part, carries a high regional diabetes prevalence at 17.6%, with the number of people with diabetes expected to increase by 92% to reach 163 million by 2050 (2). Nationally, population-based epidemiological surveys conducted in Jordan identified a high diabetes prevalence reaching up to 30% (3). Diabetic foot ulcers (DFUs) are among its most disabling and costly complications, which affect approximately 6.3% of individuals with diabetes during their lifetime (4). The transition from a simple foot ulcer to a diabetic foot infection (DFI) is a critical turning point in the disease course, with studies showing that 40-60% of diabetic foot ulcers (DFUs) will become infected (5). This transition is driven by a complex pathophysiology that involves impaired sensation caused by peripheral neuropathy, decreased perfusion as a result of peripheral arterial disease, and impaired immune response in diabetic patients (6).

This progression to DFI substantially increases the risk of morbidity and accounts for over 50% of all non-traumatic lower-limb amputations and serves as a primary reason for diabetes-related hospitalizations (5). Patients who undergo major amputation face a particularly poor prognosis. Previous research has shown that mortality following DFI is alarmingly high, with reported one- and five-year overall survival rates of 81.2% and 49.7%, respectively; in comparison, patients undergoing major amputation experienced dramatically poorer outcomes, with survival falling to 41.7% at one year and just 8.3% at five years (7).

Statins (HMG-CoA reductase inhibitors), mainstays of cardiovascular risk reduction in diabetes, possess a range of pleiotropic properties beyond their main lipid-lowering activity, that may directly counter these pathological mechanisms (8). Firstly, they directly ameliorate microvascular dysfunction, by restoring nitric oxide (NO) production, and improving endothelial function and local blood flow (9, 10). Moreover, they upregulate vascular endothelial growth factor (VEGF), actively promoting angiogenesis in ischemic tissue and improving wound healing (9, 11). As a result, statins have proved their protective effects against cardiovascular diseases in diabetic patients, and are already standard of care for reduction of cardiovascular risk (12). Statins also produced promising results in patients with other diabetes-related complications. For instance, they decreased the incidence and the need for intervention to manage diabetic retinopathy (13). Furthermore, they also showed a time-dependent renoprotective effect, by decreasing the progression of renal function decline in patients with diabetes and nephropathy (14).

In addition to their protective effects in diabetic patients, statins have been shown to have anti-inflammatory effects, as well as antibacterial potential against both gram-negative and gram-positive bacteria (15). Thus, it has been investigated in different types of infections; for example, statins effect on mortality in patients with sepsis has been extensively investigated, a meta-analysis of nine randomized clinical trials showed that their use did not improve the mortality rate in septic patients (16). In contrast, they have been associated with favorable outcomes in hospitalized patients with pneumonia (17). Overall, their effects in infectious diseases is still under debate, with inconsistent overall results.

The research on the role of statins in patients with DFI is inconsistent. Prior studies assessing the effects of statins on wound healing have produced mixed results, with some reporting no benefit (18–20), and others reporting improved healing (20, 21). In contrast, one study reported delayed recovery associated with simvastatin use (22). Moreover, investigations evaluating the effect of statins on amputation risk among diabetic patients have also yielded conflicting findings. Two studies concluded a protective effect of statins (23, 24), whereas another found that, although statins reduced the incidence of DFI, they did not decrease the need for surgical interventions, including amputation (25).

This inconsistency highlights a significant gap in real-world evidence. Additionally, no study assessed the impact of statin intensity on the amputation risk. A search of recent systematic reviews and meta-analyses did not identify studies specifically examining the association between statin therapy and amputation risk in diabetic foot disease, underscoring the novelty of the present analysis. Therefore, the present study aims to evaluate the association between statin use and the risk of amputation among patients with DFI. Secondarily, we aim to assess how the statin intensity influences amputation risk. We hypothesize that statin use will be associated with a significantly lower risk of amputation.

## 2 Methods

### 2.1 Study design and setting

This retrospective cohort study was conducted at King Abdullah University Hospital (KAUH), a tertiary referral center affiliated with the Jordan University of Science and Technology, and serves as a major referral center for northern Jordan and provides multidisciplinary diabetic foot care. The study involved a comprehensive review of electronic medical records (EMRs) for all patients who were presented with diabetic foot infections (DFIs) between January 1, 2020, and April 30, 2024.

### 2.2 Ethical considerations

The present study received approval from the Institutional Review Board (IRB) committees at King Abdullah University Hospital at the Jordan University of Science and Technology (ID#: 2019625).

### 2.3 Study population and eligibility criteria

The initial cohort consisted of all adult patients ≥ 18 years with a documented diagnosis of diabetes mellitus who were evaluated or treated for foot infection during the study period. Screening was performed using International Classification of Diseases, Tenth Revision (ICD-10) codes of diabetic foot disease.

Eligible patients were those aged ≥18 years, had a confirmed diagnosis of diabetes mellitus, met the clinical criteria for a diabetic foot infection, defined as the presence of purulent discharge or at least two classical signs of inflammation (erythema, warmth, tenderness, pain, or induration) (26). Patients were excluded if they had non-infected diabetic foot ulcers or ulcers not attributable to diabetes. Finally, to avoid misclassification bias, medical records were checked by two researchers to confirm the diagnosis.

### 2.4 Data collection

Data was extracted from EMRs using a structured and standardized data collection form. To enhance data reliability, extraction was conducted by trained research personnel.

The collected variables included demographic characteristics (age, sex, and type of diabetes), clinical parameters (most recent HbA1c level, comorbidities, and risk factors), and details of the foot infection (Wagner grade, presence of osteomyelitis confirmed radiographically, microbiologically, or clinically).

The primary predictor variable was statin therapy. Patients were classified as statin users if they had an active statin prescription within 3 months prior to admission with an active, ongoing prescription confirmed at the time of admission; no patients in this cohort had a prescription within this window that had already been discontinued by the admission date, so statin exposure reflects active use at admission rather than remote or lapsed prescribing. Statin intensity was classified according to guideline-recommended dosing. Moderate-intensity statins included atorvastatin 10–20 mg, rosuvastatin 5–10 mg, simvastatin 20–40 mg, pravastatin 40–80 mg, lovastatin 40 mg, fluvastatin XL 80 mg or 40 mg twice daily, and pitavastatin 1–4 mg daily. High-intensity statins included atorvastatin 40–80 mg and rosuvastatin 20–40 mg daily (27). In this cohort, there were no patients on low intensity statins. Statin intensity could not be determined for one patient who was confirmed to be on statin therapy but whose specific dose had been prescribed at an outside hospital and was not documented in the medical record. This patient was included in binary statin-use analyses (N = 262) but excluded from statin-intensity-stratified analyses (N = 261).

Additionally, hypertension was defined as a previously documented diagnosis or current use of antihypertensive medication. Dyslipidemia was defined as a diagnosed lipid disorder or the use of lipid-lowering therapy. Peripheral arterial disease (PAD) was defined by a documented diagnosis of PAD, symptoms of intermittent claudication, evidence of lower limb ischemia, or an ankle–brachial index (ABI) <0.9 when available.

Heart failure was defined by a recorded diagnosis or left ventricular ejection fraction <50%. Diabetic kidney disease (DKD) was defined as an estimated glomerular filtration rate <60 mL/min/1.73 m² or an albumin-to-creatinine ratio >30 mg/g persisting for more than three months. Peripheral neuropathy was defined by a documented diagnosis or absent monofilament perception at ≥2 of 10 standardized sites on either foot or diminished vibration sense (<5/8) using a 128-Hz tuning fork. Diabetic retinopathy was defined by a documented diagnosis or fundoscopic evidence of microaneurysms or more advanced retinal lesions in prior outpatient evaluations. Osteomyelitis was defined by radiographic or MRI findings consistent with infection, positive bone culture, histopathologic confirmation, or physician diagnosis. The outcome variable was amputation severity, categorized as: no amputation; minor amputation, defined as any amputation distal to the ankle joint, including toe and transmetatarsal amputations; and major amputation, defined as any amputation through or proximal to the ankle joint, including below-knee and above-knee amputations (28). Amputation outcomes were ascertained within the same hospital admission.

### 2.5 Statistical analysis

All statistical analyses were performed using SPSS software (version 27.0; IBM Corp., Armonk, NY, USA) and R software (version 4.3.1; R Foundation for Statistical Computing, Vienna, Austria). A two-sided p-value of less than 0.05 was considered statistically significant. Categorical variables were summarized as frequencies and percentages, while continuous variables were expressed, according to their distribution, as means with standard deviations (SD) or medians with interquartile ranges (IQR). Continuous variables were compared using the independent samples t-test or Mann-Whitney U test, as appropriate. Categorical variables were compared using the Chi-square test. Variables with more than 20% missing were excluded prior to imputation (**Supplementary Table 1** lists missingness by variable for excluded and imputed variables). And variables with <20% missing data were imputed using missForest package in R, which utilizes random forest imputation methods. Imputation accuracy was assessed via out-of-bag error: normalized root mean squared error (NRMSE) = 0.355 for continuous variables and proportion falsely classified (PFC) = 0.023 for categorical variables, both values indicate good imputation accuracy.

A complete-case sensitivity analysis was not performed as a formal comparative table: missingness among the covariates entering the multivariable models was limited to two variables (Wagner grade, 19.9% missing pre-imputation; diabetes control status, 9.8% missing), and restricting the sample to complete cases would preferentially exclude patients with major amputation [retaining only 37% of major amputation cases (20/54) versus 70% of the overall cohort (262/376)]. This suggests that missingness was not completely at random; a complete-case analysis would therefore itself be subject to selection bias rather than serving as an unbiased comparator.

A multivariable ordinal logistic regression model was built to assess the independent association between statin therapy and amputation severity (no amputation, minor amputation, major amputation), variables were chosen based on clinical relevance and significance in the univariate analysis. Results are presented as adjusted odds ratios (aORs) with 95% confidence intervals (CIs). The proportional odds assumption was evaluated using the Brant test for both multivariable models; the assumption was not violated overall or for any individual covariate in either Model 1 or Model 2 (all p>0.05), supporting the validity of the ordinal logistic regression approach.

Variables considered for inclusion in the multivariable models were selected based on clinical relevance and statistical significance in univariate analyses comparing patients with versus without amputation and statin users versus non-users. Age and prior history of amputation were evaluated but did not demonstrate statistical significance in either analysis and were therefore excluded from the final models. Collinearity among covariates was assessed using variance inflation factors; no evidence of problematic collinearity was identified. As the multivariable models were constructed for confounder adjustment rather than outcome prediction, discrimination statistics were not considered a primary metric of model adequacy. Propensity-score-based methods were not used in place of multivariable adjustment: statin intensity was a three-level exposure, for which propensity-score matching would require generalized propensity score methods that are less standardized than for binary exposures; additionally, the high-intensity group (n=79) and number of major amputation events (n=54) were both modest, and propensity-score matching or weighting would further reduce effective sample size and precision. Confirmed absence of problematic collinearity supported the adequacy of direct covariate adjustment as the primary confounding-control strategy.

To evaluate a dose-response relationship, the Cochran-Armitage trend test was used to assess trends in the proportions of any and major amputation across ordered statin intensity categories (none, moderate, high). To quantify the magnitude of this trend with a corresponding confidence interval, statin intensity was additionally entered as an ordinal score (0, 1, 2) in logistic regression models for major amputation and for any amputation, both unadjusted (crude trend odds ratio) and adjusted for sex, hypertension, ischemic heart disease, peripheral arterial disease, neuropathy, osteomyelitis, and Wagner grade (adjusted trend odds ratio); these factors were chosen based on the clinical importance and the statistical significance, as major amputation event rate was 54; we had a limited number of variables to include in the model.

Absolute risk reduction (ARR) and the number needed to treat (NNT) were calculated for major and minor amputation. In addition to crude absolute risk reduction (ARR) and number-needed-to-treat (NNT) calculations, adjusted risk differences were estimated using g-computation (marginal standardization). For each pairwise comparison of interest, the multivariable logistic regression model for major amputation was used to predict each patient’s outcome probability under each counterfactual exposure scenario; predicted probabilities were averaged across the cohort and differenced to obtain the adjusted risk difference. Adjusted NNT was calculated as the reciprocal of the absolute adjusted risk difference. Ninety-five percent confidence intervals for both the adjusted risk difference and adjusted NNT were obtained using a nonparametric bootstrap (2,000 resamples, percentile method).

Subgroup analyses were performed to evaluate the consistency of the association between statin use (modeled as a binary variable) and amputation severity across predefined patient subgroups. These subgroups included age, Wagner grade, and the presence of key comorbidities. For each subgroup, an ordinal logistic regression model was fitted. The results were presented as a forest plot of odds ratios with 95% CIs. In addition, formal interaction terms (statin use × subgroup) were tested for each subgroup examined to assess effect modification.

In-hospital mortality was analyzed descriptively (univariate comparison only); the small number of mortality events (6 events) precluded construction of a stable multivariable model.To identify factors associated with prolonged hospitalization, length of stay was dichotomized as prolonged (>7 days) versus standard (≤7 days). A multivariable logistic regression model was constructed with prolonged length of stay as the outcome, adjusting for statin use, Wagner grade, sex, hypertension, ischemic heart disease, stroke, peripheral arterial disease, neuropathy, uncontrolled diabetes mellitus, osteomyelitis, BMI category, and elderly age (≥65 years).

## 3 Results

### 3.1 Baseline characteristics

As shown in **Table 1**, among 376 patients with diabetic foot infection (DFI), baseline age, BMI, obesity prevalence, elderly status, and smoking rates were comparable between statin users and non-users. Male sex was more common among statin users (77% vs. 65%, p = 0.018). Statin users also had significantly higher rates of type 2 diabetes mellitus (97% vs. 91%, p = 0.009), hypertension (82% vs. 63%, p < 0.001), ischemic heart disease (47% vs. 25%, p < 0.001), stroke (22% vs. 9.6%, p = 0.004), peripheral arterial disease (45% vs. 32%, p = 0.019), and neuropathy (44% vs. 28%, p = 0.005). Other comorbidities, including heart failure, diabetic kidney disease, retinopathy, malignancy, autoimmune disease, immunosuppressant use, and chemotherapy exposure, were similar between groups.

**Table 1:** Baseline characteristics by statin intake.

Characteristic	No statin N = 114	Statin N = 262	*p*-value
Demographics			
Age, years	58 (48-70)	61 (53-69)	0.14
Male sex	74 (65%)	201 (77%)	0.018
Body Mass Index (BMI), kg/m²	28.1 (25.0-31.3)	27.7 (25.0-32.1)	>0.9
Obese (BMI >30)	45 (39.5%)	95 (36.3%)	0.55
Elderly (≥65 years)	35 (31%)	88 (34%)	0.6
Smoker	33 (29%)	80 (31%)	0.8
Comorbidities			
Type 2 Diabetes Mellitus (vs. Type 1)	104 (91%)	255 (97%)	0.009
Hypertension	72 (63%)	215 (82%)	<0.001
Ischemic Heart Disease	28 (25%)	123 (47%)	<0.001
Stroke	11 (9.6%)	58 (22%)	0.004
Atrial Fibrillation	5 (4.4%)	11 (4.2%)	>0.9
Heart Failure	26 (23%)	65 (25%)	0.7
Peripheral Arterial Disease	37 (32%)	119 (45%)	0.019
Diabetic Kidney Disease	38 (33%)	103 (39%)	0.3
Neuropathy	32 (28%)	114 (44%)	0.005
Retinopathy	33 (29%)	66 (25%)	0.4
Malignancy	3 (2.6%)	4 (1.5%)	0.4
Autoimmune disease	1 (0.9%)	3 (1.1%)	>0.9
Immunosuppressant use	5 (4.4%)	10 (3.8%)	0.8
Chemotherapy	2 (1.8%)	1 (0.4%)	0.2
Laboratory values			
Hemoglobin A1c (%)	9.10 (8.20-10.58)	8.90 (7.70-10.20)	0.3
Uncontrolled Dm (HbA1c >7)	98 (86%)	220 (84%)	0.62
Hemoglobin (g/dL)	10.20 (8.60-11.80)	10.80 (9.10-12.40)	0.061
White Blood Cell Count (×10^9^/L)	12.6 (8.8-16.6)	11.5 (8.5-14.4)	0.094
C-Reactive Protein (mg/L)	114 (62-183)	81 (38-155)	0.011
Creatinine Clearance (mL/min)	84 (52-129)	74 (48-101)	0.041
Infection and complications			
Osteomyelitis	11 (9.6%)	37 (14%)	0.2
History of Amputation	68 (60%)	128 (49%)	0.054
Type of Infection	75 (66%)	175 (67%)	0.8
Hospital course and outcomes			
Length of Stay (days)	10 (6, 15)	9 (5, 14)	0.2
Prolonged Length of Stay(>7days)	80 (70%)	162 (62%)	0.12
Amputation			0.005
No amputation	48 (42.1%)	150 (57.3%)	
Minor Amputation	41 (36.0%)	83 (31.7%)	
Major Amputation	25 (21.9%)	29 (11.1%)	
Any Amputation	66 (57.9%)	112 (42.7%)	0.007
In-Hospital Mortality	2 (1.8%)	6 (2.3%)	>0.9
Wagner grade			0.42
Grade I- III	28 (24.6%)	75 (28.6%)	
Grade IV- V	86 (75.4%)	187 (71.4%)	

One patient was confirmed to be on statin therapy but could not be classified by intensity, as the specific agent and dose had been prescribed at an outside hospital and were not documented; this patient is included in binary statin-use totals (N = 262) but excluded from statin-intensity analyses (N = 261).

Markers of glycemic control were comparable between groups. However, C-reactive protein levels were significantly higher in the no-statin group (114 vs. 81 mg/L, p = 0.011), whereas creatinine clearance was lower among statin users (74 vs. 84 mL/min, p = 0.041). Osteomyelitis rates and infection type distribution did not differ significantly between groups.

Amputation outcomes differed between cohorts. Overall, any amputation was significantly more common among non-users compared with statin users (57.9% vs. 42.7%, p = 0.007). Major amputations occurred nearly twice as often in the no-statin group (21.9% vs. 11.1%), whereas minor amputation rates were similar between groups. Median hospital stay, prolonged hospitalization, in-hospital mortality, and Wagner grade distribution were comparable between groups.

### 3.2 Regression analyses

In the multivariable ordinal regression analysis (**Table 2**), statin therapy was independently associated with lower odds of higher amputation severity after adjustment for demographic characteristics, comorbidities, diabetes-related factors, infection status, disease severity, and anthropometric measures.

**Table 2:** Multivariable ordinal regression model of amputation severity.

Variable	Model 1: aOR (95% CI)	p-value	Model 2: aOR (95% CI)	p-value
Statins				
Statin vs No statins (ref)	0.41 (0.26–0.67)	<.001	—	—
Moderate vs None (ref)	—	—	0.43 (0.20–0.74)	.004
High vs None (ref)	—	—	0.43 (0.26–0.71)	.001
Demographics				
Female vs Male (ref)	0.67 (0.40–1.10)	.112	0.68 (0.41–1.12)	.129
Comorbidities				
HTN vs No HTN (ref)	1.32 (0.78–2.21)	.297	1.32 (0.79–2.22)	.292
IHD vs No IHD (ref)	0.79 (0.50–1.24)	.299	0.79 (0.50–1.24)	.300
Stroke vs No Stroke (ref)	1.06 (0.61–1.84)	.846	1.06 (0.60–1.87)	.836
PAD vs No PAD (ref)	0.87 (0.56–1.34)	.520	0.87 (0.56–1.33)	.513
Diabetes-related				
Neuropathy vs No Neuropathy (ref)	1.50 (0.97–2.33)	.068	1.50 (0.97–2.33)	.071
Uncontrolled vs Controlled DM (ref)	0.91 (0.52–1.62)	.758	0.93 (0.52–1.65)	.807
Infection				
Osteomyelitis vs No Osteomyelitis (ref)	1.59 (0.88–2.88)	.124	1.60 (0.88–2.89)	.123
Disease severity				
High (4–5) vs Low Wagner (0–3) (ref)	6.44 (3.62–11.47)	<.001	6.47 (3.63–11.52)	<.001
Anthropometric				
Obese vs Non-obese (ref)	0.95 (0.61–1.48)	.821	0.94 (0.60–1.46)	.774

In Model 1, statin use (vs no statin use) was associated with significantly reduced odds of amputation severity (aOR 0.41, 95% CI 0.26–0.67, p < 0.001). In Model 2, when statin exposure was stratified by intensity, both moderate-intensity and high-intensity statin use were similarly associated with lower odds of amputation severity compared with no statin use (moderate-intensity: aOR 0.43, 95% CI 0.20–0.74, p = 0.004; high-intensity: aOR 0.43, 95% CI 0.26–0.71, p = 0.001).

In the adjusted model for prolonged length of stay (>7 days), only Wagner grade was significantly associated with outcome (aOR 0.35, 95% CI 0.21–0.57, p<0.001, high vs. low grade); statin use was not significantly associated with prolonged length of stay (aOR 1.37, 95% CI 0.82–2.30, p=0.232), nor was any other covariate examined (**Table 3**).

**Table 3:** Multivariable logistic regression of the association with prolonged length of stay (>7 days).

Variable	Adjusted OR (95% CI)	p-value
Wagner grade (high vs. low)	**0.35 (0.21-0.57)**	**<0.001**
BMI category	0.86 (0.54-1.38)	0.529
Uncontrolled diabetes mellitus	0.90 (0.48-1.67)	0.732
Elderly age	1.33 (0.82-2.16)	0.257
Statin use	1.37 (0.82-2.30)	0.232
Hypertension	1.13 (0.65-1.98)	0.663
Ischemic heart disease	1.05 (0.66-1.70)	0.828
Stroke	1.17 (0.66-2.08)	0.600
Neuropathy	0.70 (0.44-1.12)	0.139
Peripheral arterial disease	1.00 (0.63-1.58)	0.995
Male sex	1.19 (0.70-2.01)	0.529
Osteomyelitis	0.97 (0.48-1.94)	0.919

Bold values indicate statistical significance (p < 0.05).

### 3.3 Effect of statin intensity on major and any amputation

Effect size estimates further highlighted the clinical significance of statin therapy. Adjusted risk differences, estimated via g-computation (marginal standardization; 95% CIs via 2,000 bootstrap resamples), closely paralleled the crude ARR estimates (**Table 4**). Any statin use was associated with an adjusted risk reduction of 10.9% (95% CI 2.1–19.8%; NNT 9.2, 95% CI 5.1–47.4) for major amputation, compared with a crude ARR of 10.8% (NNT 9). Moderate-intensity statin use was associated with an adjusted risk reduction of 9.5% (95% CI 0.6–18.6%; NNT 10.5, 95% CI 5.4–173.5), and high-intensity statin use with an adjusted risk reduction of 14.4% (95% CI 4.2–24.9%; NNT 6.9, 95% CI 4.0–23.5), compared with no statin use. The direct comparison between moderate- and high-intensity statins showed no statistically significant difference (adjusted RD 4.9%, 95% CI −3.0% to 12.2%). A Cochran-Armitage trend test across the three ordered statin-intensity groups was significant for both major amputation (p=0.025) and any amputation (p=0.0039); the corresponding trend odds ratio, adjusted for sex, hypertension, ischemic heart disease, peripheral arterial disease, neuropathy, osteomyelitis, and Wagner grade, was 0.50 (95% CI 0.31–0.81) for major amputation and 0.65 (95% CI 0.47–0.91) for any amputation (**Table 5**). Together, these results indicate a statistically significant ordered trend across statin intensity groups, but do not establish that moderate- and high-intensity statins differ significantly from one another.

**Table 4:** Crude and adjusted absolute risk reduction and number needed to treat for major amputation, by statin exposure in diabetic foot infection.

Comparison	Crude ARR	Crude NNT	Adjusted RD	Adjusted RD 95% CI	Adjusted NNT	Adjusted NNT 95% CI
None vs Any statin	10.8%	9	10.9%	2.1% – 19.8%	9.2~9	5.1 – 47.4
None vs Moderate	9.3%	11	9.5%	0.6% – 18.6%	10.5~11	5.4 – 173.5
None vs High	14.3%	7	14.4%	4.2% – 24.9%	6.9~7	4.0 – 23.5
Moderate vs High	5.0%	20	4.9%	−3.0% – 12.2%	not interpretable*	—

*CI crosses zero; NNT not meaningfully interpretable.

“Adjusted risk differences estimated via g-computation (marginal standardization) using the Model 2 covariate set; 95% CIs via 2,000 bootstrap resamples (percentile method).

**Table 5:** Proportional analysis of amputation outcomes according to statin therapy intensity, with crude and adjusted trend estimates. .

Statin intensity	N (Total)	Major amputation cases	Major amputation %	Any amputation cases	Any amputation %
None	114	25	21.9%	66	57.9%
Moderate-intensity	182	23	12.6%	78	42.9%
High-intensity	79	6	7.6%	34	43.0%
Cochran–Armitage trend test results					
Trend statistic		Major amputation		Any amputation	
Cochran–Armitage trend test (p-value)		0.025		0.0039	
Crude trend OR (per 1-level increase), 95% CI		0.53 (0.34–0.81)		0.72 (0.54–0.96)	
Adjusted trend OR*, 95% CI		0.50 (0.31–0.81)		0.65 (0.47–0.91)	

*Adjusted for sex, HTN, IHD, PAD, neuropathy, osteomyelitis, and Wagner grade.

“N=182 + 79 = 261 statin users classified by intensity; one additional statin user could not be classified by intensity and is excluded here but included in **Table 4**’s ‘None vs Any statin’ row.

### 3.4 Subgroup analysis

Subgroup analyses are summarized in **Figure 1**. Across the examined subgroups, statin use appeared to be associated with a generally favorable pattern. Patients with peripheral arterial disease tended to benefit, and similar trends were seen among those without PAD. Older participants seemed to experience greater improvement compared with younger ones. The presence of neuropathy was associated with a clearer effect, though benefits were also observed in patients without neuropathy. When considering Wagner grades, the findings suggested usefulness in both less severe and more advanced lesions. Overall, the results point toward a consistent direction of benefit across vascular, neurological, and severity-based categories. However, formal tests for statin × subgroup interaction were not statistically significant for any subgroup (age: OR 1.40, 95% CI 0.57–3.42, p=0.466; Wagner grade: OR 0.67, 95% CI 0.21–2.11, p=0.496; peripheral arterial disease: OR 1.50, 95% CI 0.63–3.59, p=0.361; neuropathy: OR 1.41, 95% CI 0.58–3.47, p=0.450), providing no statistical evidence of effect modification. These subgroup findings should therefore be interpreted as exploratory and hypothesis-generating rather than confirmatory.

**Figure 1:**
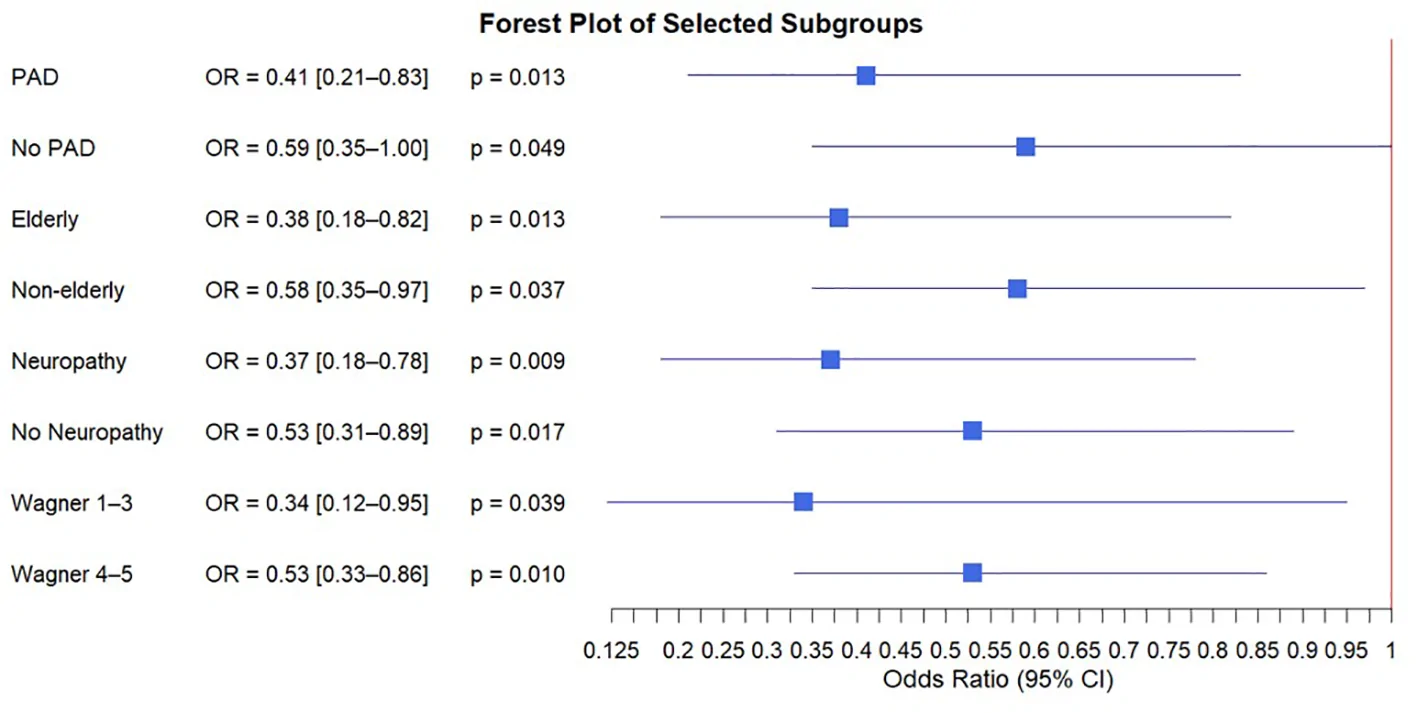
Forest plot for the association between statin use and amputation risk across clinical subgroups. Subgroup analyses shown are exploratory. Formal statin × subgroup interaction tests were non-significant for all subgroups shown (all p>0.35).

## 4 Discussion

This retrospective cohort study suggests that statin therapy is independently associated with a reduced risk of amputation, with an ordered trend across statin intensity levels that remained significant after adjustment; however, moderate- and high-intensity statins did not differ significantly from one another when compared directly. After adjustment for confounders, the estimated risk differences were 9.5% for moderate-intensity statin use and 14.4% for high-intensity therapy, corresponding to adjusted NNTs of approximately 11 and 7, respectively. As these estimates are derived from an observational cohort with potential residual confounding by indication, they should be interpreted as descriptive of the magnitude of association rather than as a guaranteed treatment effect. Furthermore, this protective effect of statins remained robust after adjustment for confounders, including Wagner grade, peripheral arterial disease, and neuropathy. The findings suggest that statin therapy, beyond its established role in cardiovascular risk reduction, may represent a potential protective strategy against limb loss in patients with diabetic foot infection.

Two prior studies have reported that statin therapy reduces the risk of amputation among patients with diabetes, findings that align with the present results (23, 24). In contrast, another study observed that while statins lowered the incidence of diabetic foot infection, they did not translate into reduced rates of surgical interventions such as amputations or angioplasty (25). Notably, none of these investigations explored the potential dose-response relationship, which our study highlights as a key and novel finding. This underscores the importance of further research to clarify whether increasing statin intensity confers progressively greater protection against limb loss.

Statins are well-known for their favorable pleiotropic effects on endothelial function, inflammation, and plaque stability, in addition to their antibacterial activity. These may explain their association with lower amputation risk in patients with DFI (8, 15, 29–31). Supporting this interpretation, patients receiving statins in our cohort had significantly lower C-reactive protein (CRP) levels, indicating reduced systemic inflammatory burden. Nonetheless, statins have been shown to increase the risk of peripheral neuropathy (32), which is a key mediator of DFI (6). Inconsistencies in previous research may be partly explained by these contrasting effects in the pathophysiological pathway of DFI.

Our results add to existing literature by evaluating the association between statin intensity and amputation risk. First, the trend analysis demonstrated a statistically significant and graded reduction in major amputation rates as statin intensity increased (p = 0.025), as reflected by a reduction in major amputation rates from 21.9% in untreated patients to 12.6%, and 7.6% in moderate- and high-intensity groups, respectively. However, this trend was driven predominantly by the difference between statin users and non-users, rather than by a graded difference between moderate- and high-intensity regimens, which did not differ significantly from one another. Notably, this gradient was significant even though the statin cohort carried a higher burden of cardiovascular comorbidities, including hypertension, ischemic heart disease, stroke, and PAD, which further highlights the strength of the observed protective association despite these differences at baseline.

The multivariable ordinal regression analysis provided further evidence that statin therapy is associated with reduced odds against progression to higher levels of amputation. In the binary-statin model, statin use was associated with substantially lower odds of severe amputation, even after adjustment for age, comorbidities, and infection severity. When stratified by intensity, both moderate- and high-intensity statins demonstrated significant protective effects, with each regimen reducing the likelihood of advancing to tissue necrosis and major amputation.

An ordered trend in amputation risk was observed across statin intensity categories, remaining statistically significant after adjustment for confounders (adjusted trend OR 0.50, 95% CI 0.31–0.81, for major amputation). However, direct comparison of moderate- and high-intensity statins showed no statistically significant difference (adjusted RD 4.9%, 95% CI −3.0% to 12.2%). This pattern supports an association between statin use and reduced amputation risk, but do not establish a true intensity-dependent gradient between moderate and high doses. In contrast, statin therapy showed no significant association with hospital length of stay or in-hospital mortality. Given the established link between amputation and long term mortality (7), extended follow-up studies are needed to determine whether the limb-salvage benefits of statins ultimately translate into improved survival outcomes.

Exploratory subgroup analyses suggested that statin therapy may be linked to reduced amputation severity across several clinically relevant categories. Formal statin × subgroup interaction tests were non-significant for every subgroup examined (age, Wagner grade, peripheral arterial disease, and neuropathy; all p≥0.35), providing no statistical evidence of true effect modification. The apparently consistent direction of benefit across subgroups should therefore not be read as confirmation of differential treatment effect, but rather as an absence of evidence against a uniform association. Larger studies will be needed to confirm whether these subgroup patterns reflect true differences.

Based on these findings, statin therapy could be considered as part of the multidisciplinary management of patients with or at high risk of diabetic foot infection. As an observational study, these findings cannot support any clinical recommendation regarding statin intensity, irrespective of the direction or significance of the moderate- versus high-intensity comparison; the association between statin use and amputation severity observed here is hypothesis-generating, and any conclusions about a comparative or intensity-dependent treatment effect, require confirmation in randomized controlled trials.

The strengths of this study include the use of detailed clinical data from electronic health records, and statistical adjustment for a comprehensive set of potential confounders. The evaluation of statin intensity as an ordered exposure and the consistent findings across subgroup analyses add depth to our conclusions, although a true intensity-dependent gradient between moderate- and high-intensity statins was not established.

However, several limitations should be acknowledged. First, the retrospective observational design precludes causal inference, and residual confounding may persist despite multivariable adjustment. Second, statin exposure was defined based on prescription records, and adherence could not be directly verified, introducing potential misclassification. Third, duration of statin therapy prior to admission and any history of prior discontinuation or re-initiation were not captured in this dataset; as such, while this study evaluated a dose-response relationship based on statin intensity, a duration-response relationship could not be assessed. Additionally, statin users differed substantially from non-users in baseline cardiovascular comorbidity burden (hypertension, ischemic heart disease, stroke, peripheral arterial disease, and neuropathy), raising the possibility of confounding by indication that may not be fully addressed by multivariable adjustment alone. Selection bias and referral bias are also possible given the tertiary-care, single-center setting, which may further limit generalizability beyond similar healthcare settings, particularly to populations outside northern Jordan.

## 5 Conclusion

This study demonstrates that statin therapy is associated with reduced amputation severity and lower risk of major amputation in patients with diabetic foot infection, with an ordered trend across statin intensity levels that remained significant after adjustment; however, moderate- and high-intensity statins did not differ significantly from one another. The association remained consistent across adjusted models and clinically relevant subgroups. However, given the retrospective design and single-center setting, multicenter randomized controlled trials are needed to confirm causality, evaluate long-term outcomes, and establish the safety of high-intensity statins in this population.

## Data Availability

The raw data supporting the conclusions of this article will be made available by the authors, without undue reservation.
