# Supplementary material for: The impact of statin therapy on amputation risk in patients with diabetic foot infection: a retrospective cohort study from Jordan
Source: Front Endocrinol (Lausanne). 2026 Sep 16;17:1922755. doi: 10.3389/fendo.2026.1922755 (PMC13623674; doi:10.3389/fendo.2026.1922755)
Supplement: Supplementary file 1 [file Table1.docx]

**Supplementary Table 1: Proportion of Missing Data and Data-Handling Decisions for Study Variables**

| **Variable** | **Missing n** | **Missing %** | **Decision** |
| --- | --- | --- | --- |
| NRBC1 | 350 | 93.1% | Excluded (>20%) |
| LDH | 342 | 91.0% | Excluded (>20%) |
| ESR | 272 | 72.3% | Excluded (>20%) |
| GGT | 215 | 57.2% | Excluded (>20%) |
| Albumin | 213 | 56.6% | Excluded (>20%) |
| ALP / ALT / AST / BBILIT / DBIL / TP | 211 each | 56.1% | Excluded (>20%) |
| LDL / TG | 198 each | 52.7% | Excluded (>20%) |
| CHOL / HDL | 192 each | 51.1% | Excluded (>20%) |
| MG | 176 | 46.8% | Excluded (>20%) |
| PHOS | 166 | 44.1% | Excluded (>20%) |
| CA | 162 | 43.1% | Excluded (>20%) |
| PTT | 123 | 32.7% | Excluded (>20%) |
| Wagner grade | 75 | 19.9% | Imputed |
| BMI | 49 | 13.0% | Imputed |
| Height | 47 | 12.5% | Imputed |
| Weight | 46 | 12.2% | Imputed |
| HbA1c | 37 | 9.8% | Imputed |
| CRP | 28 | 7.4% | Imputed |
| Creatinine | 11 | 2.9% | Imputed |
| Hemoglobin | 3 | 0.8% | Imputed |
| WBC | 3 | 0.8% | Imputed |
| All other variables | 0 | 0.0% | Complete |
